# A Comprehensive Investigation of Lipid Profile During the Solid-State Fermentation of Rice by *Monascus purpureus*

**DOI:** 10.3390/foods14030537

**Published:** 2025-02-06

**Authors:** Lan Lan, Yimin Cao, Jiajia Yuan, Rui Feng, Huiqin Pan, Xiuhong Mao, Shen Ji, Qing Hu, Heng Zhou

**Affiliations:** NMPA Key Laboratory for Quality Control of Traditional Chinese Medicine, Shanghai Institute for Food and Drug Control, Shanghai 201203, China; bluepearl1896@163.com (L.L.); tutucym@hotmail.com (Y.C.);

**Keywords:** red yeast rice, fermentation, UHPLC–Q–Orbitrap–MS, lipid analysis, medium- and long-chain triacylglycerol

## Abstract

Red yeast rice is a nutraceutical fermented product used worldwide for the symptomatic relief of dyslipidemia and cardiovascular disease. However, the fermentation-induced lipid transformation from rice to red yeast rice remains unclear. Herein, an ultra-high performance liquid chromatography coupled with hybrid quadrupole-orbitrap mass spectrometry method was developed for the comprehensive lipid analysis during fermentation. A total of 246 lipids fall in 21 subclasses were annotated in rice and red yeast rice, including 37 lysophospholipids, 14 phospholipids, 29 diglycerides, 114 triglycerides and fatty acid (15 species), ceramide (12 species), hexosylceramide (3 species), sitosterol ester (2 species), monogalactosyldiacylglycerol (2 species), digalactosyldiacylglycerol (2 species), monogalactosylmonoacylglycerol (8 species), digalactosylmonoacylglycerol (5 species), coenzyme Q (1 species), acyl hexosyl campesterol ester (1 species), and acylcarnitine (1 species). Results showed that lipid profiles changed, and new lipid species emerged. Notably, 18 medium- and long-chain triacylglycerols and triacylglycerols with short-chains were tentatively identified. These triacylglycerols also show the effects of body fat accumulation reduction, and hypolipidemic and hypoglycemic activities. Furthermore, lipid species that were profoundly changed were quantified, and the dynamic changes were investigated. This study clarified the molecular species and compositional changes in fermented rice from lipid aspect.

## 1. Introduction

Red yeast rice (RYR) is widely used as a cholesterol-lowering dietary supplement and shows significant symptomatic relief towards hypercholesterolemia and cardiovascular disease [1,2,3]. It is a nutraceutical fermented product using polished rice as the substrate, usually using *Monascus purpureus* and in rare cases with *Monascus ruber*, *Monascus pilosus,* and *Monascus anka*. The history of using RYR as food and medicine can be traced thousands of years in many Asian countries. Traditionally, RYR can be generally classified into three categories by their uses, for dyeing, for brewing, and functional RYR. Just as their names imply, RYR for dyeing and brewing are commonly used in food colorants, dyeing, and the wine-making industry [4,5]. Functional RYR is characteristic for its high-content of well-recognized, guidelines-supported metabolites, e.g., monacolin K analogs, and this study is focused on this RYR variety.

The most widely studied biosynthetic secondary metabolites in RYR are *monascus* pigments [6,7,8], statins [9,10], and saccharides [11,12], to which most of the beneficial biological and therapeutic activities are attributed, such as antidiabetic [13], anticancer [14], anti-inflammatory [15], antibacterial [16], and lipid-lowering. The citrinin pathway is also intensively studied since the unwanted mycotoxins will be produced and thus need strict monitoring [17]. Lipid analysis, from another viewpoint, provides more information for the chemical profiling of functional and nutraceutical substances in RYR. Lipids are currently intensively researched as important chemical constituents in rice. As the minor nutrient compared to starch and protein, the lipid possesses both nutritional and functional significance [18]. Furthermore, the lipid profile of rice is compared, and lipid markers are statistically obtained, which can distinguish different rice breeds and indicate grain quality changes during storage [19,20,21,22,23]. Although lipid composition in rice is relatively clear now, the dynamic change and transformation of lipid induced by fermentation is poorly understood.

Evidence has shown that rice fermentation is accompanied by the natural dissolution or leaching of lipids [24]. To reveal the fermentation process from rice to RYR in the lipidic aspect, RYR samples in different fermentation stage were individually customized. The methyl-*tert*-butyl ether (MTBE)-based lipid extraction was optimized, and an ultra-high performance liquid chromatography coupled with hybrid quadrupole-orbitrap mass spectrometry (UHPLC–Q–Orbitrap–MS) method was subsequently developed for comprehensive lipid analysis. Lipid species that were profoundly changed were quantified and their dynamic changes were described. To the best of our knowledge, this is the first investigation of lipid changes in the process of rice fermentation under *Monascus* fungi. This study would provide new insights for the molecular transformation during the fermentation-induced processing on RYR.

## 2. Materials and Methods

### 2.1. Chemical and Reagents

Palmitic acid [FA (16:0), Lot number 190029-201904], oleic acid [FA (18:1), Lot number 111621-201707] and linoleic acid [FA (18:2), Lot number 111622-200602] were obtained from the National Institutes for Food and Drug Control, China. α-linolenic acid [FA (18:3), Lot number U-62A-D3-Z] was obtained from ANPEL Laboratory Technologies (Shanghai) Inc. Shanghai, China, Avanti LightSPLASHTM LIPIDOMIX^®^ [Lot number 330732-1ML-010, containing 100 μg/mL of PC (15:0_18:1), LPC (18:1), PE (15:0_18:1), LPE (18:1), PG (15:0_18:1) (Na salt), PI (15:0_18:1) (NH_4_ salt), PS (15:0_18:1) (Na salt), TAG (15:0_18:1_15:0), DAG (15:0_18:1), MG (18:1), CE (18:1), SM (d18:1/18:1(9Z)) and Cer (d18:1/15:0)] were purchased from Sigma-Aldrich, which were produced by Avanti Polar Lipids (Alabaster, AL, USA).

LC/MS-grade acetonitrile (Merck KGaA, Darmstadt, Germany), methanol (Merck KGaA, Darmstadt, Germany), MTBE (Fisher Chemical, Geel, Belgium), isopropanol (IPA) (Merck KGaA, Darmstadt, Germany), formic acid (Fisher Scientific, Pardubice, Czech Republic), and 10 M ammonium formate solution (Sigma-Aldrich, St. Louis, MO, USA) were employed in this study. Ultrapure water was from Milli-Q system (Millipore, MA, USA). In addition, a 2 mL Safe-Lock tube (Eppendorf SE, Hamburg, Germany) and a 250 μL glass insert for a large open vial (CNW Technologies, Dusseldorf, Germany) were applied.

### 2.2. Raw and Fermented Samples

The time required for the production of RYR varies depending on the type of fermentation, inoculated strain, fermentation conditions, and the use of the RYR product. For functional RYR, fermentation time lasts from 15 days to two months. Under well-controlled industrial conditions, the *Monascus*-induced fermentation is usually accomplished within one month. In this study, individually customized fermented products at different fermentation times were used as experimental samples. A total of 200 g of polished rice was swollen with water, put into a fermentation bag, and sterilized by steaming. Inoculated with *Monascus purpureus* fungi and then cultured under proper condition by a traditional solid-state fermentation method. Samples of raw rice and rice with *Monascus* fungi were collected after drying. Moreover, fermented samples were obtained every 2 days till the 30th day, with 3 independent repetitions at each sampling time. All the rice products were stored under −20 °C avoiding light.

### 2.3. Lipids Extraction Procedures

The extraction procedure of lipids was optimized using a MTBE-based biphasic solvent system containing methanol, MTBE, and water. In brief, 50 mg of fine-ground rice and fermented products were extracted with 1 mL methanol/MTBE (1:3, *v*/*v*) for 30 min by vertical shaking with frequencies of 500 rpm (IKA-Werke GmbH & Co. KG, Staufen, Germany), followed by the mixing of 0.3 mL water. The residue was extracted repeatedly, and the upper organic phase was combined. After drying under nitrogen stream, 200 μL of acetonitrile–IPA (1:1, *v*/*v*) was used as the redissolved solvent immediately. The supernatant, after high-speed centrifugation at 14,000 rpm for 10 min (Eppendorf, Hamburg, Germany), was applied as the lipid extraction solution. Each rice product was parallelly pretreated in duplicate. The lipid extraction solution was kept under cryopreservation before injection.

### 2.4. LC–MS Analysis

The qualitative and semi-quantitative analysis of lipids were conducted on a Q–Orbitrap–MS (Thermo Fisher Scientific, Waltham, MA, USA) equipped with an electrospray ion (ESI) source, and coupled to an Agilent 1290 UHPLC–system (Agilent Technologies, Santa Clara, CA, USA) consisting of a quaternary pump, an autosampler, and a column oven.

Chromatographic separation was carried out on an ACQUITY UPLC^®^ CSH column (100 mm × 2.1 mm, 1.7 μm; Waters Corporation, Milford, MA, USA) at 55 °C with the flow rate of 0.4 mL/min. A binary mobile phase A was acetonitrile/water (60:40, *v*/*v*) with 5 mM ammonium formate and 0.1% formic acid, and B was isopropanol/acetonitrile (90:10, *v*/*v*) with 5 mM ammonium formate and 0.1% formic acid. Elution gradients were optimized as follows: 0.0–10.0 min, 2% B; 10.0–10.1 min, 2–50% B; 10.1–12.0 min, 50–54% B; 12.1–16.0 min, 54–70% B; 16.1–18.0 min, 70–99% B, 18.1–25.0 min, 2% B. The injection volume was 5 µL.

Electrospray ionization in both positive and negative ion modes was performed for the detection and the ESI parameters were set as follows: sheath gas flow rate 48 L, aux gas flow rate 11 L, sweep gas flow rate 2 L, spray voltage 2.8 KV, capillary temp 256 °C, S-lens RF level 55.0, aux gas heater temp 413 °C. Full MS/ddMS^2^ mode was applied in this study. The full scan MS parameters were as follows: mass resolution 70,000 FWHM, AGC target 3 × 10^6^, maximum IT 200 ms, full scan spectra were measured range from 50 to 1500 *m*/*z*. MS^2^ scan parameters were as follows: mass resolution 17,500 FWHM, AGC target 1 × 10^6^, maximum IT 100 ms, loop count 4, isolation window 3.0 *m*/*z*, stepped NCE were 20, 40, and 60, apex trigger 2–6 s, dynamic exclusion 5.0 s.

### 2.5. Quality Assurance

To guarantee the accuracy and stability of the instrument, the mass spectrometer was calibrated using Thermo Tune software Version 2.8 SP1 Build 2806 (Thermo Fisher Scientific), and QC samples were intermittently injected into every six samples throughout the whole batch. A total of 50 µL of each test sample was pooled together to obtain the QC sample.

### 2.6. Statistical Analysis

The lipid molecular species were identified based on chromatographic and mass spectrometry information, according to the accurate mass and fragment matching using LipidSearch Software Version 5.1 (Thermo Fisher Scientific) and the online LIPID MAPS database. The top four peaks from each MS raw file were manually and processed using Thermo TraceFinder 5.1 General Quan 2.0 (Thermo Fisher Scientific). The contents of lipid species from rice to red yeast rice during the 30-days’ fermentation were quantitatively evaluated using an external calibration method established by lipid standards of the same or similar categories. Wherein FA (16:0) is applied for the quantification of FA (16:0), FA (14:0) and FA (16:1), FA (18:1) for FA (18:1) and FA (18:0), LPC (18:1) for LPCs, LPE (18:1) for LPEs, LPGs, AcCa (2:0) and CoQ10, MG (18:1) for MGMG and DGMG, and TAG (15:0_18:1_15:0) for the quantification of TAGs. The calibration curves are displayed in Table S2.

MS data were loaded into Progenesis QI software Version 2.4.6911.27652 (Waters Corporation, Manchester, UK). Based on the identified lipids, the embedded EZinfo 2.0 software (Waters Corporation, Milford, MA, USA) was used for further multivariate data matrix processing.

## 3. Results and Discussion

### 3.1. Optimization of the Lipid Extraction

As a fundamental and essential step in lipid analytical procedures, lipid extraction exerts a major influence on lipid coverage and data reliability. Usually, the lipid extraction from rice can be carried out using CHCl_3_-based and MTBE-based extraction solvent systems [19,20,22,23]. Considering the lower density of MTBE and methanol, when the upper lipid-rich organic layer is transferred and collected, it does not need to pass through the aqueous phase and the insoluble substances at the interface between these two phases, which can reduce the contamination and simplify the pretreatment during lipid extraction. Apart from that, the hazards to the human body and the environment are also a concern. Consequently, MTBE-based lipid extraction was further optimized in this study.

The rations of methanol, MTBE, and water altered in the experimental design for the optimization of MTBE method. The extraction efficiencies were evaluated by the total ion intensity acquired on the mass spectrometer and the detectable peak capacity. The TIC of the optimized extraction solution under positive and negative ionization mode are given in Figure S1. The preferred solvent system was MeOH/MTBE (1:3, *v*/*v*, 1 mL) combined with 0.3 mL water owing to the larger peak capacity and higher MS response. The extraction of raw rice and fermented rice is shown in Figure S2. It was observed that a darker-reddish color appeared in the lower aqueous phase, which indicated that some water-soluble impurities such as *Monascus* pigments were removed through the liquid–liquid separation.

### 3.2. Lipid Identification by UHPLC–Q–Orbitrap–MS

Mechanistic studies of lipid fragmentation pathways have been carried out for a wide range of lipid species under the collision-induced dissociation. In the present study, Cer, HexCer, and LPE tend to render the percussor ion of [M + H]^+^, TAG, DAG, and the SiE produce precursor ion of [M + NH_4_]^+^ under positive ESI ionization mode. Glycerol glycolipids such as MGMG, DGMG, MGDG, and DGDG form primarily [M + HCOO]^−^ under ESI^–^, as well as PC. FA and several phospholipids (PL) are recognized by the occurrence of [M − H]^−^ parent ion. These lipid molecules also produce fragment ions under specific patterns. Cer and HexCer would dehydrate continuously and generate ion products of [M + H − nH_2_O]^+^. DAG and TAG yield fragment ions attribute to the loss of ammonia combined with the neutral loss of FA chains. Depending on the acquisition mode polarity, PL render fragments of headgroups, FA moieties, or both as ions and neutrals. Thirteen lipids in Avanti LightSPLASHTM LIPIDOMIX^®^ also provide evidence of fragmentation patterns for the structural elucidation.

**Figure 1:**
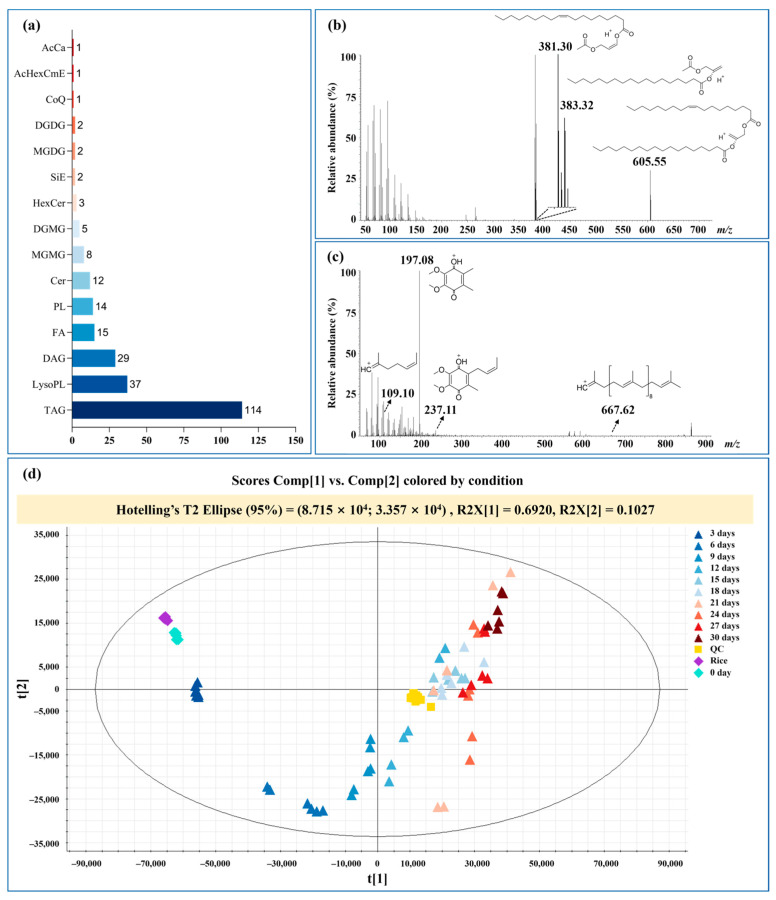
Lipids identified in rice and RYR (**a**); representative fragmentation of TAG (2:0_18:0_18_1) (**b**) and CoQ10 (**c**); PCA score plot of the fermentation process under ESI^+^ (**d**). Zero day represents raw rice with fungi; Different color represents rice sample at different fermentation stage.

The representative MS spectra under both ESI^+^ and ESI^–^ ionization modes are exhibited in Figure S3. TAG and DAG contribute the most abundant responses under positive ionization mode. In total, six classes, and 21 subclasses of 246 lipids were identified in raw and fermented rice products (Table S1). The annotated lipids are TAG (46%), LysoPL (15%), DAG (12%), FA (6%), PL (6%), Cer (5%), MGMG (3%), DGMG (2%), Hexcer (1%), SiE (1%), MGDG (1%), DGDG (1%), CoQ (0.4%), AcHexCmE (0.4%), and AcCa (0.4%), as shown in Figure 1a. The derivation of precursor ion and product ion of TAG (2:0_18:0_18_1) and CoQ10 were displayed in Figure 1b,c.

According to the established LC–MS analysis method, the MS raw data of each rice sample were collected. The PCA score plot under the positive ionization mode was obtained using the multivariate data analysis, which could be used to evaluate the overall influence of *Monascus*-induced fermentation on the lipidic spectrum, as shown in Figure 1d. The result demonstrated that the lipid profiles of RYR were obviously separated from the raw rice, and clearly showed the trend of lipid changes during the whole fermentation process. To monitor the performance of the data acquisition system, QC samples in the entire injection procedure were measured. The result suggested that all QCs were tightly clustered, which would guarantee stability and reproducibility in the whole analysis of the system.

### 3.3. TAG Profile Changed in Fermentation Process

TAG is a major source of stored energy in animals and the major constituent of plant oils [25,26]. According to the length of the FA carbon chain, TAG can be divided into medium-chain triacylglycerol (MCT), medium- and long-chain triacylglycerol (MLCT), and long-chain triacylglycerol (LCT). Medium-chain FAs are the types of FA that contain 6 to 12 carbon atoms, and long-chain FAs contain more than 12 carbon atoms. TAG with one or two medium-chain FAs, and at least one long-chain FA is classified as MLCT. It is a structured lipid with both medium- and long-chain FAs in one TAG molecule. FAs with a chain length shorter than 14 and longer than 22 carbon atoms are present only in minor concentrations.

MLCT possesses different physicochemical properties, metabolic characteristics, and nutritional values. It not only provides us with the essential fatty acids, more importantly, it also reduces body fat accumulation, improves insulin resistance, and plays an important role in clinical nutritional treatment [27,28,29]. The natural sources of MLCT are mammalian milk fat and their products, and a small amount exists in tropical oils. Common oils and fats mainly comprise LCT [30,31]. Due to the limited natural sources of MLCT, plant oils are used as raw materials in industrial preparation by enzymatic and chemical synthesis for high purity MLCT with specific molecular structures [32].

**Figure 2:**
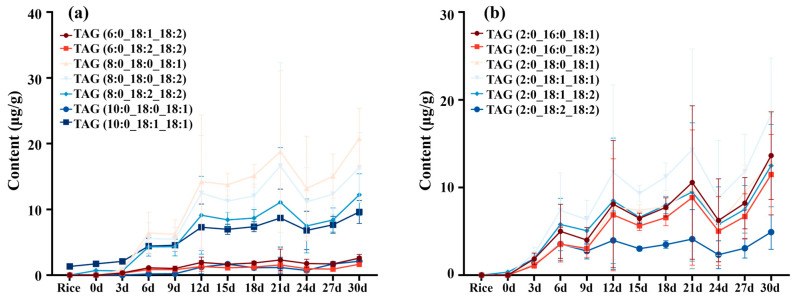
The changes in TAG in fermented rice (**a**) MLCTs and (**b**) TAGs with SCFA. Data are expressed as the mean ± standard deviation.

A large change in the TAG profile was observed, and MLCT was biosynthesized in the fermentation process (Figure 2a). Medium-chain FAs in these newly emerged MLCTs were caproic acid (6:0) and caprylic acid (8:0), mainly coupling with long-chain FAs such as stearic acid (18:0), oleic acid (18:1 n-9), and linoleic acid (18:2 n-6). The contents of TAG (8:0_18:0_18:1), TAG (8:0_18:0_18:2), and TAG (8:0_18:2_18:2) reached 20.78 ± 4.60, 16.34 ± 3.32, and 12.23 ± 3.18 μg/g, respectively, at the 30th day. The content of MLCT with capric acid (10:0) moiety also significantly increased, e.g., TAG (10:0_18:1_18:1) increased by seven-fold after fermentation for 30 days.

In this study, TAGs with an even shorter FA chain, such as the FA 2:0 moiety, were found to be newly generated during fermentation (Figure 2b). Short-chain FAs (SCFA) have been less discussed than medium- or long- chain FAs. However, increasing evidence indicates the importance of SCFAs in regulating hypertension, ischaemic reperfusion, myocardial infarction, atherosclerosis, and heart failure [33]. The amounts of TAG (2:0_16:0_18:1), TAG (2:0_16:0_18:2), TAG (2:0_18:0_18:1), TAG (2:0_18:1_18:1), TAG (2:0_18:1_18:2), and TAG (2:0_18:2_18:2) were 13.63 ± 4.98, 11.48 ± 2.57, 13.01 ± 4.12, 18.32 ± 4.47, 12.49 ± 4.70, and 4.91 ± 1.96 μg/g at the 30th day, respectively. The TAG contents shared the same variation trend. They continuously increased till day 21 and reached their peak on day 30.

Research has been conducted to elucidate the potential lipid-lowering compounds other than monacolins in RYR [34]. Here, we provided a series of MLCTs, and TAGs with short-chain FAs, which would expand the scope of active ingredients in RYR because of the lipid regulation effect they might present [35,36,37]. These characterized TAGs were displayed in Table 1.

### 3.4. Phospholipids Changed in Fermentation Process

Lipids in rice endosperm are present in different forms compared to those in the grain and germs, and these molecules are classified as starch and non-starch lipids. Starch lipids are complexes in which lipids are associated with starch granules in the endosperm [38]. Apart from free FAs, the major starch lipids are LPC and LPE, which account for about 50% of the starch lipid in non-waxy rice [39]. The study of mass spectrometry imaging on rice has shown that LPC species spread throughout the tissue [40]. The hypothesis originally proposed that these conjunct lipids would release, along with the consumption of amylose by *Monascus*. However, the transformation of PL during the fermentation is more complicated than we expected.

**Figure 3:**
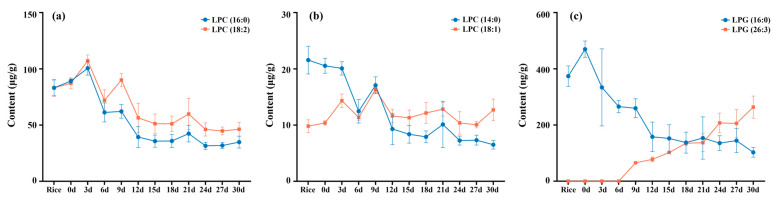
The changes in LPC (**a**,**b**) and LPG (**c**) in fermented rice.

As shown in Figure 3a,b, the contents of LPC 14:0 (elution time 2.39 min), 16:0 (elution time 4.08 min) and 18:2 (elution time 3.22 min), though they increased in the first 3–9 days, eventually decreased by two–three-fold on day 30. The detections of LPC 18:1 were approximately unchanged. In the observation of the PL subclass with lower content, LPG (26:3) possessing a polyunsaturated FA chain was emerging, and its content was 263.45 ± 39.36 ng/g on day 30 (Figure 3c). QC samples throughout the entire injections showed that several abundant LPEs were not stable enough to render the variation trend, and the results were not displayed in this study.

### 3.5. Other Lipids Changed in Fermentation Process

**Figure 4:**
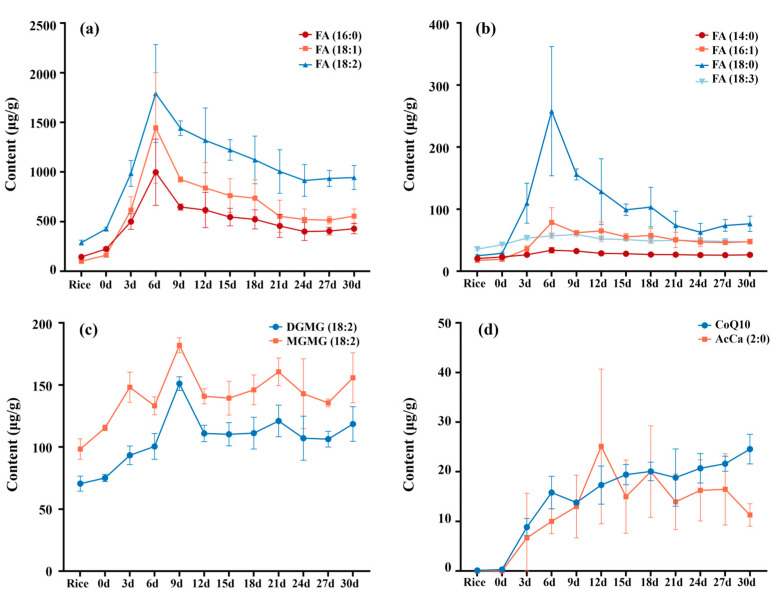
The changes in FA (**a**,**b**), DGMG and MGMG (**c**), and CoQ10 and AcCa (2:0) (**d**) in fermented rice.

To further assess the lipid transformation during fermentation, the quantification of more lipids belonging to different subclasses was investigated. FA is a substantial part of a lipid, and is an important energy substrate, which plays multiple roles in humans and other organisms. As shown in Figure 4a, the contents of free FAs, such as palmitic (16:0), oleic (18:1), and linoleic (18:2) acid were relatively abundant. Their amounts were 224.00 ± 17.59, 161.80 ± 6.10, and 426.45 ± 15.17 μg/g on day 0, increased to the highest 997.25 ± 233.95, 1444.35 ± 256.99, and 1790.16 ± 392.18 μg/g on day 6, then decreased to 429.24 ± 51.35, 555.74 ± 71.16, and 944.03 ± 121.09 μg/g on day 30, respectively. FA (14:0), FA (16:1), FA (18:0), and FA (18:3) also showed the same variation pattern, which reached the peak values on day 6, followed by a slight decline on days 9–30 (Figure 4b). Ultimately, the FA content nearly doubled during the 30-days’ fermentation.

The detections of two substantial galactolipids with an unsaturated FA chain, DGMG (18:2) and MGMG (18:2), are shown in Figure 4c. The values reached their maximum on day 9, which were 151.04 ± 5.49 μg/g for DGMG (18:2) and 181.85 ± 6.08 μg/g for MGMG (18:2). The contents dropped to 118.51 ± 13.99 and 155.71 ± 20.14 μg/g on day 30, which was much higher than that on day 0. Although it might be partially relevant, the composition of lipids and the content of MGMG in the cell membrane was related to the integrity of the cell membrane, which would influence the biosynthesis and secretion of *Monascus* pigments in the current study [41].

Coenzyme Q10 (CoQ10) is one of the most important metabolites of *Monascus*. It is a lipophilic vitamin-like antioxidant that is present in nearly all the tissues. It is essential for converting energy from carbohydrates and fatty acids to adenosine triphosphate in mitochondrial energy metabolism, and is responsible for a multitude of physiological functions. CoQ10 in fermented rice can also enforce the effectiveness of statins and reduce their side effects [42]. CoQ10 was found to be continuously rising from 0.12 to 24.56 ± 2.98 μg/g in the fermentation period. AcCa (2:0), biosynthesizing mainly through glycolysis, pyruvate metabolism, and tricarboxylic acid cycle, varied greatly from 0 (day 0), 25.11 ± 10.59 (day 12), 20.02 ± 9.23 (day 18) to 11.29 ± 2.27 (day 30) μg/g (Figure 4d). AcCa (2:0) is essential for the transport of LCFAs from the cytoplasm to the mitochondrial matrix, where FA oxidation occurs [43]. It also acts as the starter unit in the biosynthesis of *monascus* pigments and greatly enhances under the metabolism of carbohydrates [44].

### 3.6. Lipid Biosynthesis Regulation for Monascus-Fermented Rice

Lipid mechanistic regulation for *Monascus*-fermented rice tends to be influenced by different external stimuli and stresses. It is reported that in response to the imbalance of cell homeostasis, the regulation mechanism of lipids was implemented [45,46]. However, the functional annotation to the most significantly changed lipid molecules is insufficient, which makes the metabolic pathway in the process of *Monascus*-fermentation difficult to discover. Here, in this study, the changes in lipid profiles were thoroughly described through absolute quantitation, which hopefully provides more information for lipid biosynthesis regulation during the *Monascus*-induced fermentation.

## 4. Conclusions

Lipid analysis based on an UHPLC–Q–Orbitrap–MS method was conducted on fermented rice by inoculating *Monascus* within 30 days. Altogether, 246 lipid species belonging to 21 subclasses were annotated in raw and fermented rice products. It is notable that different subclasses of lipids showed differentiated fluctuation trends in the 30-days’ fermentation period, which might involve the process of firstly either transformation or synthesis, and then reuse and consumption with *Monascus*. More importantly, 18 molecules of MLCT and TAG coupling with a short carbon chain emerged, which may provide new prospects in the discovery of lipid-lowering active substances in RYR. Also, although the fermentation condition and management were kept the same to the maximum, the measurements of lipids still occasionally presented large deviations, which indicated the fermentation process was not always synchronized. This study clarified the molecular species and compositional changes in lipid aspects during the fermentation-induced processing on red yeast rice, which provides useful information for *Monascus*-fermented rice.

## Figures and Tables

**Table 1:** Identification of TAGs with medium- chain FAs and short-chain FAs.

No.	Lipid Species	Formula	RT (min)	Proposed Ion	Observed *m*/*z*	Theoretical *m*/*z*	Fragmental Ions (Measured)
1	TAG (O-14:2_4:0_18:2)	C_39_H_68_O_5_	16.72	[M + NH_4_]^+^	634.5385	634.5405	337.2734, 599.5027, 617.5138
2	TAG (2:0_18:2_18:2)	C_41_H_70_O_6_	17.11	[M + NH_4_]^+^	676.5496	676.5511	379.2839, 599.5024
3	TAG (2:0_16:0_18:2)	C_39_H_70_O_6_	17.92	[M + NH_4_]^+^	652.5495	652.5511	379.2839, 355.2839, 575.5029
4	TAG (2:0_18:1_18:2)	C_41_H_72_O_6_	17.94	[M + NH_4_]^+^	678.5649	678.5667	381.2995, 379.2841, 601.5186
5	TAG (O-13:1_5:0_18:1)	C_39_H_72_O_5_	18.38	[M + NH_4_]^+^	638.5699	638.5718	339.2892, 603.5346
6	TAG (6:0_18:2_18:2)	C_45_H_78_O_6_	18.79	[M + NH_4_]^+^	732.6116	732.6137	435.3466, 599.5028
7	TAG (2:0_16:0_18:1)	C_39_H_72_O_6_	18.80	[M + NH_4_]^+^	654.5652	654.5667	381.2995, 355.2838, 577.5183
8	TAG (2:0_18:1_18:1)	C_41_H_74_O_6_	18.84	[M + NH_4_]^+^	680.5808	680.5824	381.2995, 603.5334
9	TAG (6:0_18:1_18:2)	C_45_H_80_O_6_	19.32	[M + NH_4_]^+^	734.6268	734.6293	437.3624, 435.3468, 601.5186
10	TAG (8:0_18:2_18:2)	C_47_H_82_O_6_	19.37	[M + NH_4_]^+^	760.6427	760.6450	463.3776, 599.5026
11	TAG (2:0_18:0_18:1)	C_41_H_76_O_6_	19.41	[M + NH_4_]^+^	682.5964	682.5980	381.2997, 383.3152, 605.5497
12	TAG (O-15:0_18:0_3:0)	C_39_H_76_O_5_	19.57	[M + NH_4_]^+^	642.6016	642.6031	341.3048, 607.5656
13	TAG (8:0_16:0_18:2)	C_45_H_82_O_6_	19.65	[M + NH_4_]^+^	736.6432	736.6450	463.3775, 439.3776, 575.5028
14	TAG (8:0_18:1_18:2)	C_47_H_84_O_6_	19.70	[M + NH_4_]^+^	762.6589	762.6606	465.3937, 463.3778, 601.5184
15	TAG (8:0_18:0_18:2)	C_47_H_86_O_6_	19.88	[M + NH_4_]^+^	764.6741	764.6763	603.5343, 467.4092, 463.3776
16	TAG (10:0_18:1_18:1)	C_49_H_90_O_6_	20.05	[M + NH_4_]^+^	792.7055	792.7076	493.4249, 603.5344
17	TAG (8:0_18:0_18:1)	C_47_H_88_O_6_	20.05	[M + NH_4_]^+^	766.6901	766.6919	605.5496, 467.4090, 465.3934
18	TAG (10:0_18:0_18:1)	C_49_H_92_O_6_	20.31	[M + NH_4_]^+^	794.7205	794.7232	495.4403, 493.4248, 605.5496

## Data Availability

The original contributions presented in the study are included in the article/Supplementary Material, further inquiries can be directed to the corresponding authors.

## Supplementary Materials

The following supporting information can be downloaded at: https://www.mdpi.com/article/10.3390/foods14030537/s1, Figure S1: TIC of optimized RYR extraction solution in positive ionization mode (a) and negative ionization mode (b); Figure S2: Extraction of rice products based on MTBE method; Table S1: Annotated lipids in rice products by UHPLC–Q–Orbitrap–MS; Table S2: Calibrations used in the quantification of lipids in rice product.

## Abbreviations

The following abbreviations are used in this manuscript:

AcCa	Acylcarnitine
AcHexCmE	Acyl hexosyl campesterol ester
Cer	Ceramide
CoQ	Coenzyme Q
DAG	Diacylglycerol
DGDG	Digalactosyldiacylglycerol
DGMG	Digalactosylmonoacylglycerol
FA	Fatty acid
HexCer	Hexosylceramide
LCT	Long-chain triacylglycerol
LPC	Lysophosphatidylcholine
LPE	Lysophosphatidylethanolamine
LPG	Lysophosphatidylglycerol
LPI	Lysophosphatidylinositol
MLCT	Medium- and long-chain triacylglycerol
MCT	Medium-chain triacylglycerol
MTBE	Methyl-*tert*-butyl ether
MGDG	Monogalactosyldiacylglycerol
MGMG	Monogalactosylmonoacylglycerol
NIFDC	National Institutes for Food and Drug Control
PC	Phosphatidylcholine
PE	Phosphatidylethanolamine
PG	Phosphatidylglycerol
PI	Phosphatidylinositol
PL	Phospholipid
SCFA	Short-chain fatty acid
SiE	Sitosterol ester
TAG	Triacylglycerol
