# Supplementary material for: A Comprehensive Investigation of Lipid Profile During the Solid-State Fermentation of Rice by Monascus purpureus
Source: Foods. 2025 Feb 6;14(3):537. doi: 10.3390/foods14030537 (PMC11817215; doi:10.3390/foods14030537)
Supplement: Supplementary file 1 [file foods-14-00537-s001.zip › foods-3440454-supplementary.pdf]

A Comprehensive Investigation of Lipid Profile During the Solid–state Fermentation of Rice by *Monascus Purpureus*

Lan Lan †, Yimin Cao †, Jiajia Yuan, Rui Feng, Huiqin Pan, Xiuhong Mao, Shen Ji, Qing Hu *, Heng Zhou *

NMPA Key Laboratory for Quality Control of Traditional Chinese Medicine, Shanghai Institute for Food and Drug Control, Shanghai 201203, China

* Correspondence: Qing Hu, huqingyjslw@163.com; Heng Zhou, shadowless@126.com; Tel.: +86-21-50798195

† These authors contributed equally to this work

Table S1

Annotated lipids in rice products by UHPLC–Q–Orbitrap–MS.

No.	Lipid species	RT (min)	Adduct	Ion Formula	observed m/z	Theoretical m/z	Subclass
1	AcCa(2:0)	1.22	$[M+H]^+$	$C_9H_{18}NO_4$	204.1227	204.1230	AcCa
2	LPG(14:0)	2.05	$[M–H]^-$	$C_{20}H_{40}O_9P$	455.2415	455.2415	LPG
3	LPE(14:0)	2.17	$[M–H]^-$	$C_{19}H_{39}NO_7P$	424.2470	424.2470	LPE
4	LPC(14:0)	2.19	$[M+HCOO]^-$	$C_{23}H_{47}NO_9P$	512.2991	512.2994	LPC
5	LPG(14:0)	2.19	$[M–H]^-$	$C_{20}H_{40}O_9P$	455.2415	455.2415	LPG
6	LPE(18:2)	2.21	$[M+H]^+$	$C_{23}H_{45}NO_7P$	478.2920	478.2928	LPE
7	LPE(18:3)	2.24	$[M–H]^-$	$C_{23}H_{41}NO_7P$	474.2624	474.2626	LPE
8	LPC(18:3)	2.26	$[M+HCOO]^-$	$C_{27}H_{49}NO_9P$	562.3148	562.3150	LPC
9	LPE(14:0)	2.34	$[M–H]^-$	$C_{19}H_{39}NO_7P$	424.2470	424.2470	LPE
10	LPI(18:2)	2.34	$[M–H]^-$	$C_{27}H_{48}O_{12}P$	595.2889	595.2889	LPI
11	LPC(14:0)	2.37	$[M+HCOO]^-$	$C_{23}H_{47}NO_9P$	512.2991	512.2994	LPC
12	LPE(18:3)	2.39	$[M–H]^-$	$C_{23}H_{41}NO_7P$	474.2624	474.2626	LPE
13	LPC(18:3)	2.42	$[M+HCOO]^-$	$C_{27}H_{49}NO_9P$	562.3148	562.3150	LPC
14	LPG(18:2)	2.66	$[M–H]^-$	$C_{24}H_{44}O_9P$	507.2725	507.2728	LPG
15	LPC(16:1)	2.68	$[M+HCOO]^-$	$C_{25}H_{49}NO_9P$	538.3155	538.3150	LPC
16	DGMG(18:2)	2.73	$[M+HCOO]^-$	$C_{34}H_{59}O_{16}$	723.3820	723.3809	DGMG
17	LPI(16:0)	2.77	$[M–H]^-$	$C_{25}H_{48}O_{12}P$	571.2896	571.2889	LPI
18	LPE(18:2)	2.88	$[M–H]^-$	$C_{23}H_{43}NO_7P$	476.2782	476.2783	LPE
19	LPG(18:2)	2.90	$[M–H]^-$	$C_{24}H_{44}O_9P$	507.2725	507.2728	LPG
20	LPC(18:2)	2.91	$[M+HCOO]^-$	$C_{27}H_{51}NO_9P$	564.3305	564.3307	LPC
21	LPE(18:1)	2.93	$[M+H]^+$	$C_{23}H_{47}NO_7P$	480.3078	480.3085	LPE

22	DGMG(18:2)	3.01	$[M+HCOO]^-$	$C_{34}H_{59}O_{16}$	723.3820	723.3809	DGMG
23	LPE(18:2)	3.14	$[M+H]^+$	$C_{23}H_{45}NO_7P$	478.2919	478.2928	LPE
24	LPE(18:2)	3.14	$[M–H]^-$	$C_{23}H_{43}NO_7P$	476.2779	476.2783	LPE
25	LPC(18:2)	3.18	$[M+HCOO]^-$	$C_{27}H_{51}NO_9P$	564.3302	564.3307	LPC
26	LPG(16:0)	3.20	$[M–H]^-$	$C_{22}H_{44}O_9P$	483.2724	483.2728	LPG
27	MGMG(14:0)	3.30	$[M+HCOO]^-$	$C_{24}H_{45}O_{11}$	509.2968	509.2967	MGMG
28	DGMG(16:0)	3.32	$[M+HCOO]^-$	$C_{32}H_{59}O_{16}$	699.3807	699.3809	DGMG
29	LPC(16:0)	3.56	$[M+HCOO]^-$	$C_{25}H_{51}NO_9P$	540.3305	540.3307	LPC
30	LPG(16:0)	3.57	$[M–H]^-$	$C_{22}H_{44}O_9P$	483.2725	483.2728	LPG
31	DGMG(16:0)	3.77	$[M+HCOO]^-$	$C_{32}H_{59}O_{16}$	699.3819	699.3809	DGMG
32	LPG(16:0)	3.94	$[M–H]^-$	$C_{22}H_{44}O_9P$	483.2726	483.2728	LPG
33	LPE(16:0)	3.94	$[M–H]^-$	$C_{21}H_{43}NO_7P$	452.2780	452.2783	LPE
34	LPG(16:0)	3.99	$[M–H]^-$	$C_{22}H_{44}O_9P$	483.2726	483.2728	LPG
35	LPC(16:0)	3.99	$[M+HCOO]^-$	$C_{25}H_{51}NO_9P$	540.3303	540.3307	LPC
36	LPE(18:1)	4.01	$[M–H]^-$	$C_{23}H_{45}NO_7P$	478.2941	478.2939	LPE
37	LPG(18:1)	4.03	$[M–H]^-$	$C_{24}H_{46}O_9P$	509.2884	509.2885	LPG
38	LPE(18:1)	4.13	$[M–H]^-$	$C_{23}H_{45}NO_7P$	478.2941	478.2939	LPE
39	LPG(18:1)	4.16	$[M–H]^-$	$C_{24}H_{46}O_9P$	509.2884	509.2885	LPG
40	LPC(18:1)	4.18	$[M+HCOO]^-$	$C_{27}H_{53}NO_9P$	566.3465	566.3463	LPC
41	MGMG(18:2)	4.25	$[M+HCOO]^-$	$C_{28}H_{49}O_{11}$	561.3282	561.3280	MGMG
42	DGMG(18:1)	4.38	$[M+HCOO]^-$	$C_{34}H_{61}O_{16}$	725.3978	725.3965	DGMG
43	LPE(18:1)*	4.61	$[M–H]^-$	$C_{23}H_{45}NO_7P$	478.2941	478.2939	LPE
44	LPC(18:1)*	4.68	$[M+HCOO]^-$	$C_{27}H_{53}NO_9P$	566.3465	566.3463	LPC
45	MGMG(18:2)	4.70	$[M+HCOO]^-$	$C_{28}H_{49}O_{11}$	561.3282	561.3280	MGMG
46	MGMG(16:0)	4.75	$[M+HCOO]^-$	$C_{26}H_{49}O_{11}$	537.3285	537.3280	MGMG
47	MGMG(16:0)	5.49	$[M+HCOO]^-$	$C_{26}H_{49}O_{11}$	537.3285	537.3280	MGMG

48	MGMG(16:0)	5.53	$[M+HCOO]^-$	$C_{26}H_{49}O_{11}$	537.3285	537.3280	MGMG
49	MGMG(16:0)	6.25	$[M+HCOO]^-$	$C_{26}H_{49}O_{11}$	537.3283	537.3280	MGMG
50	MGMG(18:1)	7.43	$[M+HCOO]^-$	$C_{28}H_{51}O_{11}$	563.3441	563.3437	MGMG
51	LPC(18:0)	7.86	$[M+HCOO]^-$	$C_{27}H_{55}NO_9P$	568.3625	568.3620	LPC
52	Cer(d18:0_2:0)	7.88	$[M+H]^+$	$C_{20}H_{42}NO_3$	344.3157	344.3159	Cer
53	FA(18:3)*	12.70	$[M–H]^-$	$C_{18}H_{29}O_2$	277.2173	277.2173	FA
54	FA(14:0)	12.88	$[M–H]^-$	$C_{14}H_{27}O_2$	227.2018	227.2017	FA
55	FA(16:1)	13.15	$[M–H]^-$	$C_{16}H_{29}O_2$	253.2172	253.2173	FA
56	DAG(18:1_26:7)	13.18	$[M+NH_4]^+$	$C_{47}H_{80}NO_5$	738.6037	738.6031	DAG
57	Cer(d20:0_2:0)	13.34	$[M+H]^+$	$C_{22}H_{46}NO_3$	372.3465	372.3472	Cer
58	FA(18:2)*	13.50	$[M–H]^-$	$C_{18}H_{31}O_2$	279.2327	279.2330	FA
59	FA(17:1)	13.79	$[M–H]^-$	$C_{17}H_{31}O_2$	267.2327	267.2330	FA
60	FA(16:0)*	14.18	$[M–H]^-$	$C_{16}H_{31}O_2$	255.2328	255.2330	FA
61	TAG(18:1_18:2_18:2)	14.28	$[M+NH_4]^+$	$C_{57}H_{104}NO_6$	898.7828	898.7858	TAG
62	TAG(16:1_18:1_18:2)	14.28	$[M+NH_4]^+$	$C_{55}H_{102}NO_6$	872.7676	872.7702	TAG
63	FA(18:1)*	14.33	$[M–H]^-$	$C_{18}H_{33}O_2$	281.2483	281.2486	FA
64	TAG(16:1_18:1_18:2)	14.41	$[M+NH_4]^+$	$C_{55}H_{102}NO_6$	872.7676	872.7702	TAG
65	TAG(18:1_18:2_18:2)	14.41	$[M+NH_4]^+$	$C_{57}H_{104}NO_6$	898.7828	898.7858	TAG
66	FA(17:0)	14.62	$[M–H]^-$	$C_{17}H_{33}O_2$	269.2487	269.2486	FA
67	PE(18:2_18:2)	14.88	$[M–H]^-$	$C_{41}H_{73}NO_8P$	738.5083	738.5079	PE
68	FA(20:1)	14.92	$[M–H]^-$	$C_{20}H_{37}O_2$	309.2800	309.2799	FA
69	FA(18:0)	14.95	$[M–H]^-$	$C_{18}H_{35}O_2$	283.2641	283.2643	FA
70	DAG(8:0_18:2)	14.96	$[M+NH_4]^+$	$C_{29}H_{56}NO_5$	498.4138	498.4153	DAG
71	TAG(14:0_16:0_18:2)	14.97	$[M+NH_4]^+$	$C_{51}H_{98}NO_6$	820.7358	820.7389	TAG
72	TAG(14:0_16:0_18:2)	15.02	$[M+NH_4]^+$	$C_{51}H_{98}NO_6$	820.7358	820.7389	TAG
73	TAG(14:0_16:0_18:2)	15.10	$[M+NH_4]^+$	$C_{51}H_{98}NO_6$	820.7358	820.7389	TAG

74	TAG(16:1_16:1_18:1)	15.18	$[M+NH_4]^+$	$C_{53}H_{100}NO_6$	846.7514	846.7545	TAG
75	TAG(14:0_16:0_18:2)	15.18	$[M+NH_4]^+$	$C_{51}H_{98}NO_6$	820.7358	820.7389	TAG
76	TAG(16:0_18:2_18:2)	15.31	$[M+NH_4]^+$	$C_{55}H_{102}NO_6$	872.7676	872.7702	TAG
77	PI(16:0_18:2)	15.38	$[M–H]^-$	$C_{43}H_{78}O_{13}P$	833.5194	833.5186	PI
78	PI(18:1_18:2)	15.40	$[M–H]^-$	$C_{45}H_{80}O_{13}P$	859.5346	859.5342	PI
79	PE(16:0_16:0)	15.41	$[M–H]^-$	$C_{37}H_{73}NO_8P$	690.5083	690.5079	PE
80	TAG(14:0_16:0_18:2)	15.45	$[M+NH_4]^+$	$C_{51}H_{98}NO_6$	820.7358	820.7389	TAG
81	FA(20:0)	15.51	$[M–H]^-$	$C_{20}H_{39}O_2$	311.2956	311.2956	FA
82	PG(16:0_16:0)	15.56	$[M–H]^-$	$C_{38}H_{74}O_{10}P$	721.5029	721.5025	PG
83	DGDG(18:2_18:2)	15.57	$[M+HCOO]^-$	$C_{52}H_{89}O_{17}$	985.6112	985.6105	DGDG
84	PC(18:2_18:2)	15.57	$[M+HCOO]^-$	$C_{45}H_{81}NO_{10}P$	826.5609	826.5604	PC
85	TAG(14:0_16:0_18:2)	15.61	$[M+NH_4]^+$	$C_{51}H_{98}NO_6$	820.7358	820.7389	TAG
86	TAG(18:1_18:2_18:2)	15.62	$[M+NH_4]^+$	$C_{57}H_{104}NO_6$	898.7828	898.7858	TAG
87	TAG(16:0_18:2_18:2)	15.62	$[M+NH_4]^+$	$C_{55}H_{102}NO_6$	872.7676	872.7702	TAG
88	PI(16:0_18:1)	15.76	$[M–H]^-$	$C_{43}H_{80}O_{13}P$	835.5355	835.5342	PI
89	Cer(d18:2_18:2)	15.77	$[M+HCOO]^-$	$C_{37}H_{66}NO_5$	604.4954	604.4946	Cer
90	PI(16:0_18:1)	15.79	$[M–H]^-$	$C_{43}H_{80}O_{13}P$	835.5355	835.5342	PI
91	PG(16:0_16:0)	15.85	$[M–H]^-$	$C_{38}H_{74}O_{10}P$	721.5029	721.5025	PG
92	TAG(18:1_18:2_18:2)	15.85	$[M+NH_4]^+$	$C_{57}H_{104}NO_6$	898.7826	898.7858	TAG
93	TAG(16:0_18:2_18:2)	15.85	$[M+NH_4]^+$	$C_{55}H_{102}NO_6$	872.7673	872.7702	TAG
94	TAG(14:0_16:0_18:2)	15.88	$[M+NH_4]^+$	$C_{51}H_{98}NO_6$	820.7355	820.7389	TAG
95	TAG(18:0_18:0_18:2)	15.91	$[M+NH_4]^+$	$C_{57}H_{110}NO_6$	904.8289	904.8328	TAG
96	MGDG(18:2_18:2)	15.94	$[M+HCOO]^-$	$C_{46}H_{79}O_{12}$	823.5591	823.5577	MGDG
97	DGDG(16:0_18:2)	16.00	$[M+HCOO]^-$	$C_{50}H_{89}O_{17}$	961.6114	961.6105	DGDG
98	PC(16:0_18:2)	16.01	$[M+HCOO]^-$	$C_{43}H_{81}NO_{10}P$	802.5611	802.5604	PC
99	PC(18:1_18:2)	16.02	$[M+HCOO]^-$	$C_{45}H_{83}NO_{10}P$	828.5768	828.5760	PC

100	HexCer(d18:2_18:2)	16.05	$[M+H]^+$	$C_{42}H_{76}NO_8$	722.5534	722.5565	HexCer
101	TAG(14:0_16:0_18:2)	16.05	$[M+NH_4]^+$	$C_{51}H_{98}NO_6$	820.7355	820.7389	TAG
102	TAG(18:1_18:2_18:2)	16.05	$[M+NH_4]^+$	$C_{57}H_{104}NO_6$	898.7826	898.7858	TAG
103	TAG(16:0_18:2_18:2)	16.06	$[M+NH_4]^+$	$C_{55}H_{102}NO_6$	872.7673	872.7702	TAG
104	DAG(16:2_18:2)	16.10	$[M+NH_4]^+$	$C_{37}H_{68}NO_5$	606.5077	606.5092	DAG
105	PE(16:0_18:2)	16.20	$[M–H]^-$	$C_{39}H_{73}NO_8P$	714.5088	714.5079	PE
106	DAG(18:2_18:3)	16.20	$[M+NH_4]^+$	$C_{39}H_{70}NO_5$	632.5232	632.5249	DAG
107	FA(22:0)	16.21	$[M–H]^-$	$C_{22}H_{43}O_2$	339.3264	339.3269	FA
108	Cer(d19:2_18:2)	16.22	$[M+H]^+$	$C_{37}H_{68}NO_3$	574.5177	574.5194	Cer
109	HexCer(d19:2_18:2)	16.22	$[M+H]^+$	$C_{43}H_{78}NO_8$	736.5705	736.5722	HexCer
110	Cer(d18:2_16:0)	16.22	$[M+H]^+$	$C_{34}H_{66}NO_3$	536.5023	536.5037	Cer
111	HexCer(d19:2_18:2)	16.26	$[M+H]^+$	$C_{43}H_{78}NO_8$	736.5705	736.5722	HexCer
112	TAG(16:0_18:2_18:2)	16.27	$[M+NH_4]^+$	$C_{55}H_{102}NO_6$	872.7673	872.7702	TAG
113	TAG(18:1_18:2_18:2)	16.28	$[M+NH_4]^+$	$C_{57}H_{104}NO_6$	898.7826	898.7858	TAG
114	TAG(18:1_18:1_18:3)	16.37	$[M+NH_4]^+$	$C_{57}H_{104}NO_6$	898.7826	898.7858	TAG
115	TAG(16:0_18:1_18:3)	16.37	$[M+NH_4]^+$	$C_{55}H_{102}NO_6$	872.7673	872.7702	TAG
116	TAG(18:0_18:0_18:2)	16.37	$[M+NH_4]^+$	$C_{57}H_{110}NO_6$	904.8289	904.8328	TAG
117	DAG(17:2_18:2)	16.39	$[M+NH_4]^+$	$C_{38}H_{70}NO_5$	620.5230	620.5249	DAG
118	Cer(d19:2_18:1)	16.49	$[M+H]^+$	$C_{37}H_{70}NO_3$	576.5336	576.5350	Cer
119	PC(16:0_16:0)	16.52	$[M+HCOO]^-$	$C_{41}H_{81}NO_{10}P$	778.5611	778.5604	PC
120	PC(16:0_18:1)	16.54	$[M+HCOO]^-$	$C_{43}H_{83}NO_{10}P$	804.5764	804.5760	PC
121	DAG(14:0_18:2)	16.55	$[M+NH_4]^+$	$C_{35}H_{68}NO_5$	582.5079	582.5092	DAG
122	DAG(14:0_18:2)	16.61	$[M+NH_4]^+$	$C_{35}H_{68}NO_5$	582.5079	582.5092	DAG
123	DAG(16:1_18:2)	16.61	$[M+NH_4]^+$	$C_{37}H_{70}NO_5$	608.5235	608.5249	DAG
124	DAG(16:1_18:2)	16.64	$[M+NH_4]^+$	$C_{37}H_{70}NO_5$	608.5235	608.5249	DAG
125	Cer(d19:2_18:2)	16.67	$[M+H]^+$	$C_{37}H_{68}NO_3$	574.5177	574.5194	Cer

126	TAG(O-14:2_4:0_18:2)	16.72	$[M+NH_4]^+$	$C_{39}H_{72}NO_5$	634.5385	634.5405	TAG
127	Cer(d19:2_18:2)	16.73	$[M+H]^+$	$C_{37}H_{68}NO_3$	574.5177	574.5194	Cer
128	MGDG(16:0_16:0)	16.73	$[M+HCOO]^-$	$C_{42}H_{79}O_{12}$	775.5585	775.5577	MGDG
129	TAG(16:0_18:2_18:2)	16.96	$[M+NH_4]^+$	$C_{55}H_{102}NO_6$	872.7681	872.7702	TAG
130	TAG(18:1_18:1_18:3)	16.96	$[M+NH_4]^+$	$C_{57}H_{104}NO_6$	898.7836	898.7858	TAG
131	DAG(17:1_18:2)	17.02	$[M+NH_4]^+$	$C_{38}H_{72}NO_5$	622.5391	622.5405	DAG
132	Cer(d19:2_18:1)	17.03	$[M+H]^+$	$C_{37}H_{70}NO_3$	576.5336	576.5350	Cer
133	TAG(2:0_18:2_18:2)	17.11	$[M+NH_4]^+$	$C_{41}H_{74}NO_6$	676.5496	676.5511	TAG
134	TAG(18:0_18:2_18:3)	17.11	$[M+NH_4]^+$	$C_{57}H_{104}NO_6$	898.7836	898.7858	TAG
135	FA(24:0)	17.12	$[M-H]^-$	$C_{24}H_{47}O_2$	367.3585	367.3582	FA
136	TAG(17:1_18:1_18:2)	17.14	$[M+NH_4]^+$	$C_{56}H_{104}NO_6$	886.7841	886.7858	TAG
137	Cer(d18:2_18:0)	17.14	$[M+H]^+$	$C_{36}H_{70}NO_3$	564.5340	564.5350	Cer
138	TAG(15:0_18:1_18:2)	17.14	$[M+NH_4]^+$	$C_{54}H_{102}NO_6$	860.7683	860.7702	TAG
139	DAG(14:0_18:1)	17.20	$[M+NH_4]^+$	$C_{35}H_{70}NO_5$	584.5238	584.5249	DAG
140	DAG(14:0_18:1)	17.27	$[M+NH_4]^+$	$C_{35}H_{70}NO_5$	584.5238	584.5249	DAG
141	DAG(16:0_16:1)	17.30	$[M+NH_4]^+$	$C_{35}H_{70}NO_5$	584.5238	584.5249	DAG
142	DAG(14:0_18:1)	17.34	$[M+NH_4]^+$	$C_{35}H_{70}NO_5$	584.5238	584.5249	DAG
143	TAG(18:0_18:2_18:3)	17.38	$[M+NH_4]^+$	$C_{57}H_{104}NO_6$	898.7829	898.7858	TAG
144	DAG(16:0_18:2)	17.43	$[M+NH_4]^+$	$C_{37}H_{72}NO_5$	610.5388	610.5405	DAG
145	DAG(18:1_18:2)	17.46	$[M+NH_4]^+$	$C_{39}H_{74}NO_5$	636.5539	636.5562	DAG
146	DAG(18:1_18:2)	17.49	$[M+NH_4]^+$	$C_{39}H_{74}NO_5$	636.5539	636.5562	DAG
147	TAG(15:0_18:1_18:2)	17.49	$[M+NH_4]^+$	$C_{54}H_{102}NO_6$	860.7672	860.7702	TAG
148	TAG(17:1_18:1_18:2)	17.50	$[M+NH_4]^+$	$C_{56}H_{104}NO_6$	886.7828	886.7858	TAG
149	Cer(d18:2_20:1)	17.62	$[M+H]^+$	$C_{38}H_{72}NO_3$	590.5494	590.5507	Cer
150	Cer(d18:2_20:1)	17.71	$[M+H]^+$	$C_{38}H_{72}NO_3$	590.5494	590.5507	Cer

151	DAG(15:0_18:1)	17.78	$[M+NH_4]^+$	$C_{36}H_{72}NO_5$	598.5393	598.5405	DAG
152	DAG(18:1_18:2)	17.79	$[M+NH_4]^+$	$C_{39}H_{74}NO_5$	636.5549	636.5562	DAG
153	DAG(17:1_18:1)	17.84	$[M+NH_4]^+$	$C_{38}H_{74}NO_5$	624.5546	624.5562	DAG
154	DAG(15:0_18:1)	17.84	$[M+NH_4]^+$	$C_{36}H_{72}NO_5$	598.5393	598.5405	DAG
155	TAG(2:0_16:0_18:2)	17.92	$[M+NH_4]^+$	$C_{39}H_{74}NO_6$	652.5495	652.5511	TAG
156	TAG(2:0_18:1_18:2)	17.94	$[M+NH_4]^+$	$C_{41}H_{76}NO_6$	678.5649	678.5667	TAG
157	DAG(17:0_18:2)	17.96	$[M+NH_4]^+$	$C_{38}H_{74}NO_5$	624.5546	624.5562	DAG
158	FA(26:0)	18.31	$[M-H]^-$	$C_{26}H_{51}O_2$	395.2893	395.3895	FA
159	DAG(16:0_18:1)	18.36	$[M+NH_4]^+$	$C_{37}H_{74}NO_5$	612.5546	612.5562	DAG
160	TAG(17:0_18:1_18:3)	18.37	$[M+NH_4]^+$	$C_{56}H_{104}NO_6$	886.7832	886.7858	TAG
161	TAG(O-13:1_5:0_18:1)	18.38	$[M+NH_4]^+$	$C_{39}H_{76}NO_5$	638.5699	638.5718	TAG
162	DAG(18:0_18:2)	18.55	$[M+NH_4]^+$	$C_{39}H_{76}NO_5$	638.5699	638.5718	DAG
163	TAG(6:0_18:2_18:2)	18.79	$[M+NH_4]^+$	$C_{45}H_{82}NO_6$	732.6116	732.6137	TAG
164	TAG(2:0_16:0_18:1)	18.80	$[M+NH_4]^+$	$C_{39}H_{76}NO_6$	654.5652	654.5667	TAG
165	TAG(2:0_16:0_18:1)	18.84	$[M+NH_4]^+$	$C_{39}H_{76}NO_6$	654.5652	654.5667	TAG
166	TAG(2:0_18:1_18:1)	18.84	$[M+NH_4]^+$	$C_{41}H_{78}NO_6$	680.5808	680.5824	TAG
167	DAG(17:0_18:1)	18.85	$[M+NH_4]^+$	$C_{38}H_{76}NO_5$	626.5703	626.5718	DAG
168	DAG(17:0_18:1)	18.89	$[M+NH_4]^+$	$C_{38}H_{76}NO_5$	626.5703	626.5718	DAG
169	TAG(14:0_16:0_18:1)	18.99	$[M+NH_4]^+$	$C_{51}H_{100}NO_6$	822.7522	822.7545	TAG
170	TAG(16:0_18:1_18:2)	19.11	$[M+NH_4]^+$	$C_{55}H_{104}NO_6$	874.7827	874.7858	TAG
171	DAG(16:0_18:0)	19.15	$[M+NH_4]^+$	$C_{37}H_{76}NO_5$	614.5698	614.5718	DAG
172	TAG(16:0_18:1_18:2)	19.17	$[M+NH_4]^+$	$C_{55}H_{104}NO_6$	874.7827	874.7858	TAG
173	DAG(18:0_18:1)	19.18	$[M+NH_4]^+$	$C_{39}H_{78}NO_5$	640.5855	640.5875	DAG
174	TAG(18:1_18:2_20:2)	19.19	$[M+NH_4]^+$	$C_{59}H_{108}NO_6$	926.8127	926.8171	TAG
175	TAG(16:0_16:0_18:2)	19.19	$[M+NH_4]^+$	$C_{53}H_{102}NO_6$	848.7682	848.7702	TAG

176	TAG(18:1_18:1_18:2)	19.19	$[M+NH_4]^+$	$C_{57}H_{106}NO_6$	900.7981	900.8015	TAG
177	FA(28:0)	19.21	$[M–H]^–$	$C_{28}H_{55}O_2$	423.4210	423.4208	FA
178	TAG(16:0_18:1_18:2)	19.25	$[M+NH_4]^+$	$C_{55}H_{104}NO_6$	874.7827	874.7858	TAG
179	TAG(18:1_18:1_18:2)	19.25	$[M+NH_4]^+$	$C_{57}H_{106}NO_6$	900.7981	900.8015	TAG
180	TAG(18:1_20:1_18:3)	19.32	$[M+NH_4]^+$	$C_{59}H_{108}NO_6$	926.8127	926.8171	TAG
181	TAG(6:0_18:1_18:2)	19.32	$[M+NH_4]^+$	$C_{45}H_{84}NO_6$	734.6268	734.6293	TAG
182	TAG(8:0_18:2_18:2)	19.37	$[M+NH_4]^+$	$C_{47}H_{86}NO_6$	760.6427	760.6450	TAG
183	TAG(18:1_18:1_18:2)	19.37	$[M+NH_4]^+$	$C_{57}H_{106}NO_6$	900.7981	900.8015	TAG
184	TAG(16:0_16:0_18:2)	19.37	$[M+NH_4]^+$	$C_{53}H_{102}NO_6$	848.7682	848.7702	TAG
185	TAG(2:0_18:0_18:1)	19.41	$[M+NH_4]^+$	$C_{41}H_{80}NO_6$	682.5964	682.5980	TAG
186	TAG(18:1_20:1_18:3)	19.43	$[M+NH_4]^+$	$C_{59}H_{108}NO_6$	926.8127	926.8171	TAG
187	TAG(18:0_18:2_18:2)	19.43	$[M+NH_4]^+$	$C_{57}H_{106}NO_6$	900.7981	900.8015	TAG
188	TAG(15:0_16:0_18:1)	19.57	$[M+NH_4]^+$	$C_{52}H_{102}NO_6$	836.7679	836.7702	TAG
189	TAG(20:0_18:1_18:1)	19.57	$[M+NH_4]^+$	$C_{59}H_{114}NO_6$	932.8608	932.8641	TAG
190	TAG(O-15:0_18:0_3:0)	19.57	$[M+NH_4]^+$	$C_{39}H_{80}NO_5$	642.6016	642.6031	TAG
191	DAG(20:0_18:1)	19.60	$[M+NH_4]^+$	$C_{41}H_{82}NO_5$	668.6176	668.6188	DAG
192	TAG(16:0_17:0_18:2)	19.60	$[M+NH_4]^+$	$C_{54}H_{104}NO_6$	862.7838	862.7858	TAG
193	TAG(18:0_18:0_18:1)	19.60	$[M+NH_4]^+$	$C_{57}H_{112}NO_6$	906.8462	906.8484	TAG
194	TAG(8:0_16:0_18:2)	19.65	$[M+NH_4]^+$	$C_{45}H_{86}NO_6$	736.6432	736.6450	TAG
195	TAG(10:0_18:2_18:2)	19.67	$[M+NH_4]^+$	$C_{49}H_{90}NO_6$	788.6742	788.6763	TAG
196	AcHexCmE(16:0)	19.68	$[M+NH_4]^+$	$C_{50}H_{92}NO_7$	818.6844	818.6868	AcHexCmE
197	TAG(8:0_18:1_18:2)	19.70	$[M+NH_4]^+$	$C_{47}H_{88}NO_6$	762.6589	762.6606	TAG
198	TAG(18:0_20:0_18:2)	19.71	$[M+NH_4]^+$	$C_{59}H_{114}NO_6$	932.8608	932.8641	TAG
199	TAG(22:0_18:1_18:2)	19.72	$[M+NH_4]^+$	$C_{61}H_{116}NO_6$	958.8763	958.8797	TAG
200	TAG(24:0_18:2_18:2)	19.74	$[M+NH_4]^+$	$C_{63}H_{118}NO_6$	984.8925	984.8954	TAG

201	TAG(18:0_20:0_18:2)	19.77	$[M+NH_4]^+$	$C_{59}H_{114}NO_6$	932.8608	932.8641	TAG
202	TAG(16:0_18:1_18:1)	19.77	$[M+NH_4]^+$	$C_{55}H_{106}NO_6$	876.7987	876.8015	TAG
203	TAG(18:1_20:1_18:2)	19.78	$[M+NH_4]^+$	$C_{59}H_{110}NO_6$	928.8293	928.8328	TAG
204	TAG(16:0_16:0_18:1)	19.81	$[M+NH_4]^+$	$C_{53}H_{104}NO_6$	850.7840	850.7858	TAG
205	TAG(16:0_18:0_18:2)	19.85	$[M+NH_4]^+$	$C_{55}H_{106}NO_6$	876.7987	876.8015	TAG
206	TAG(18:1_18:0_18:2)	19.86	$[M+NH_4]^+$	$C_{57}H_{108}NO_6$	902.8140	902.8171	TAG
207	TAG(8:0_16:0_18:1)	19.87	$[M+NH_4]^+$	$C_{45}H_{88}NO_6$	738.6581	738.6606	TAG
208	TAG(8:0_18:0_18:2)	19.88	$[M+NH_4]^+$	$C_{47}H_{90}NO_6$	764.6741	764.6763	TAG
209	TAG(10:0_18:1_18:2)	19.88	$[M+NH_4]^+$	$C_{49}H_{92}NO_6$	790.6896	790.6919	TAG
210	TAG(18:1_18:1_18:1)	19.89	$[M+NH_4]^+$	$C_{57}H_{108}NO_6$	902.8140	902.8171	TAG
211	TAG(12:0_18:2_18:2)	19.91	$[M+NH_4]^+$	$C_{51}H_{94}NO_6$	816.7049	816.7076	TAG
212	TAG(20:0_18:2_18:2)	19.93	$[M+NH_4]^+$	$C_{59}H_{110}NO_6$	928.8293	928.8328	TAG
213	TAG(16:1_18:2_18:3)	19.95	$[M+NH_4]^+$	$C_{55}H_{98}NO_6$	868.7368	868.7389	TAG
214	TAG(14:0_18:2_18:3)	19.96	$[M+NH_4]^+$	$C_{53}H_{96}NO_6$	842.7211	842.7232	TAG
215	TAG(18:2_18:2_18:3)	19.96	$[M+NH_4]^+$	$C_{57}H_{100}NO_6$	894.7524	894.7545	TAG
216	TAG(24:0_18:1_18:1)	19.98	$[M+NH_4]^+$	$C_{63}H_{122}NO_6$	988.9234	988.9267	TAG
217	SiE(18:2)	19.98	$[M+NH_4]^+$	$C_{47}H_{84}NO_2$	694.6477	694.6497	SiE
218	CoQ10	20.01	$[M+H]^+$	$C_{59}H_{91}O_4$	863.6896	863.6912	Co
219	TAG(16:0_17:0_18:1)	20.01	$[M+NH_4]^+$	$C_{54}H_{106}NO_6$	864.7991	864.8015	TAG
220	TAG(16:0_18:2_17:3)	20.03	$[M+NH_4]^+$	$C_{54}H_{98}NO_6$	856.7365	856.7389	TAG
221	SiE(18:2)	20.04	$[M+NH_4]^+$	$C_{47}H_{84}NO_2$	694.6477	694.6497	SiE
222	TAG(18:0_24:0_18:2)	20.04	$[M+NH_4]^+$	$C_{63}H_{122}NO_6$	988.9234	988.9267	TAG
223	TAG(10:0_18:1_18:1)	20.05	$[M+NH_4]^+$	$C_{49}H_{94}NO_6$	792.7055	792.7076	TAG
224	TAG(8:0_18:0_18:1)	20.05	$[M+NH_4]^+$	$C_{47}H_{92}NO_6$	766.6901	766.6919	TAG
225	TAG(14:0_18:2_18:2)	20.07	$[M+NH_4]^+$	$C_{53}H_{98}NO_6$	844.7369	844.7389	TAG
226	TAG(18:1_18:2_18:3)	20.10	$[M+NH_4]^+$	$C_{57}H_{102}NO_6$	896.7672	896.7702	TAG

227	TAG(16:0_18:2_18:3)	20.11	$[M+NH_4]^+$	$C_{55}H_{100}NO_6$	870.7525	870.7545	TAG
228	TAG(18:1_17:2_18:3)	20.13	$[M+NH_4]^+$	$C_{56}H_{100}NO_6$	882.7518	882.7545	TAG
229	TAG(18:0_18:1_18:1)	20.13	$[M+NH_4]^+$	$C_{57}H_{110}NO_6$	904.8295	904.8328	TAG
230	TAG(16:0_16:0_18:0)	20.17	$[M+NH_4]^+$	$C_{53}H_{106}NO_6$	852.7987	852.8015	TAG
231	TAG(16:0_17:2_18:2)	20.20	$[M+NH_4]^+$	$C_{54}H_{100}NO_6$	858.7523	858.7545	TAG
232	TAG(18:0_18:0_18:2)	20.20	$[M+NH_4]^+$	$C_{57}H_{110}NO_6$	904.8295	904.8328	TAG
233	TAG(24:0_18:1_18:2)	20.21	$[M+NH_4]^+$	$C_{63}H_{120}NO_6$	986.9072	986.9110	TAG
234	TAG(20:0_18:1_18:2)	20.21	$[M+NH_4]^+$	$C_{59}H_{112}NO_6$	930.8451	930.8484	TAG
235	TAG(16:0_16:0_18:0)	20.24	$[M+NH_4]^+$	$C_{53}H_{106}NO_6$	852.7987	852.8015	TAG
236	TAG(24:0_18:1_18:2)	20.25	$[M+NH_4]^+$	$C_{63}H_{120}NO_6$	986.9072	986.9110	TAG
237	TAG(17:1_18:1_18:3)	20.26	$[M+NH_4]^+$	$C_{56}H_{102}NO_6$	884.7679	884.7702	TAG
238	TAG(22:0_18:2_18:2)	20.26	$[M+NH_4]^+$	$C_{61}H_{114}NO_6$	956.8612	956.8641	TAG
239	TAG(20:0_18:1_18:2)	20.26	$[M+NH_4]^+$	$C_{59}H_{112}NO_6$	930.8451	930.8484	TAG
240	TAG(24:0_18:1_18:2)	20.30	$[M+NH_4]^+$	$C_{63}H_{120}NO_6$	986.9072	986.9110	TAG
241	TAG(20:0_18:1_18:2)	20.30	$[M+NH_4]^+$	$C_{59}H_{112}NO_6$	930.8451	930.8484	TAG
242	TAG(22:0_18:2_18:2)	20.30	$[M+NH_4]^+$	$C_{61}H_{114}NO_6$	956.8612	956.8641	TAG
243	TAG(10:0_18:0_18:1)	20.31	$[M+NH_4]^+$	$C_{49}H_{96}NO_6$	794.7205	794.7232	TAG
244	TAG(14:0_16:0_18:2)	20.32	$[M+NH_4]^+$	$C_{51}H_{98}NO_6$	820.7364	820.7389	TAG
245	TAG(16:1_18:1_18:2)	20.32	$[M+NH_4]^+$	$C_{55}H_{102}NO_6$	872.7679	872.7702	TAG
246	TAG(18:1_18:2_18:2)	20.35	$[M+NH_4]^+$	$C_{57}H_{104}NO_6$	898.7831	898.7858	TAG

* Further confirmed with reference compounds.

Table S2

Calibrations used in the quantification of lipids in rice product.

Standards	Regression curve	Calibration for	r^2	r
FA (16:0)	$y=6.976e^5X-2.706e^6$	FA (14:0), FA (16:0), FA (16:1)	0.9980	0.9990
FA (18:1)	$y=1.640e^6X-5.556e^6$	FA (18:0), FA (18:1)	0.9992	0.9996
FA (18:2)	$y=2.091e^6X-8.381e^6$	FA (18:2)	0.9988	0.9994
FA (18:3)	$y=1.523e^6X-5.222e^6$	FA (18:3)	0.9991	0.9995
LPC (18:1)	$y=5.161e^8X-1.003e^7$	LPC	0.9984	0.9992
LPE (18:1)	$y=1.419e^8X-2.142e^6$	LPE, LPG, AcCa (2:0), CoQ10	0.9991	0.9995
MG (18:1)	$y=1.760e^6X-3.787e^5$	MGMG, DGMG	0.9970	0.9985
TG (15:0_18:1_15:0)	$y=1.620e^8X-2.219e^6$	TG	0.9997	0.9998

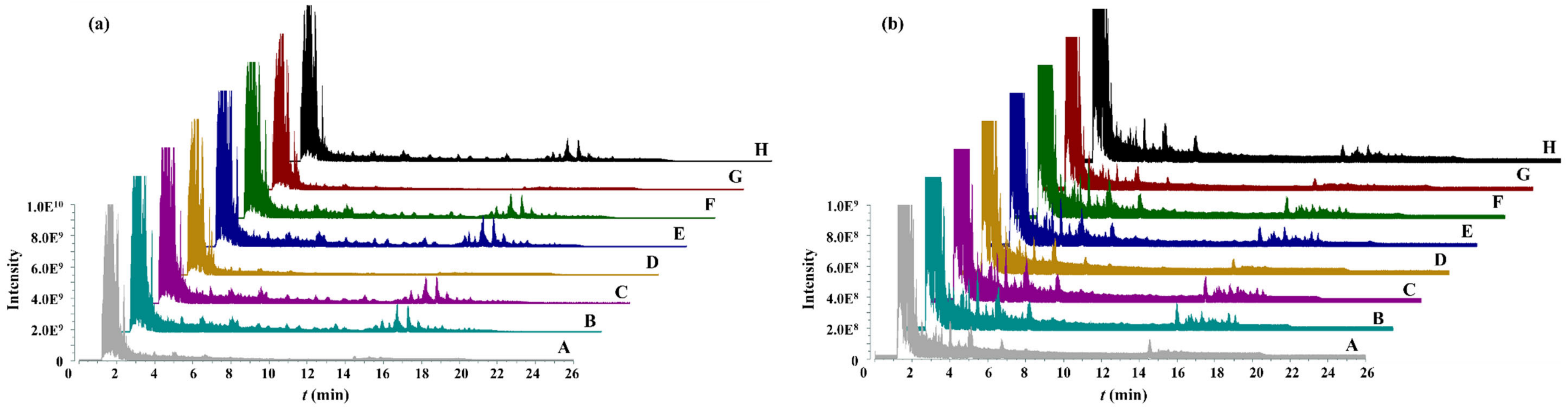

Figure S1. TIC of optimized RYR extraction solution in positive ionization mode (a) and negative ionization mode (b). A MeOH/MTBE (1:3, *v*/*v*, 1ml) + H_2O (0.5 ml); B MeOH/MTBE (1:3, *v*/*v*, 1ml) + H_2O (0.4 ml); C MeOH/MTBE (1:3, *v*/*v*, 1ml) + H_2O (0.3 ml); D MeOH/MTBE (1:3, *v*/*v*, 1ml) + H_2O (0.2 ml); E MeOH/MTBE (1:2, *v*/*v*, 1ml) + H_2O (0.5 ml); F MeOH/MTBE (1:2, *v*/*v*, 1ml) + H_2O (0.4 ml); G MeOH/MTBE (1:2, *v*/*v*, 1ml) + H_2O (0.3 ml); H MeOH/MTBE (1:2, *v*/*v*, 1ml) + H_2O (0.2 ml).

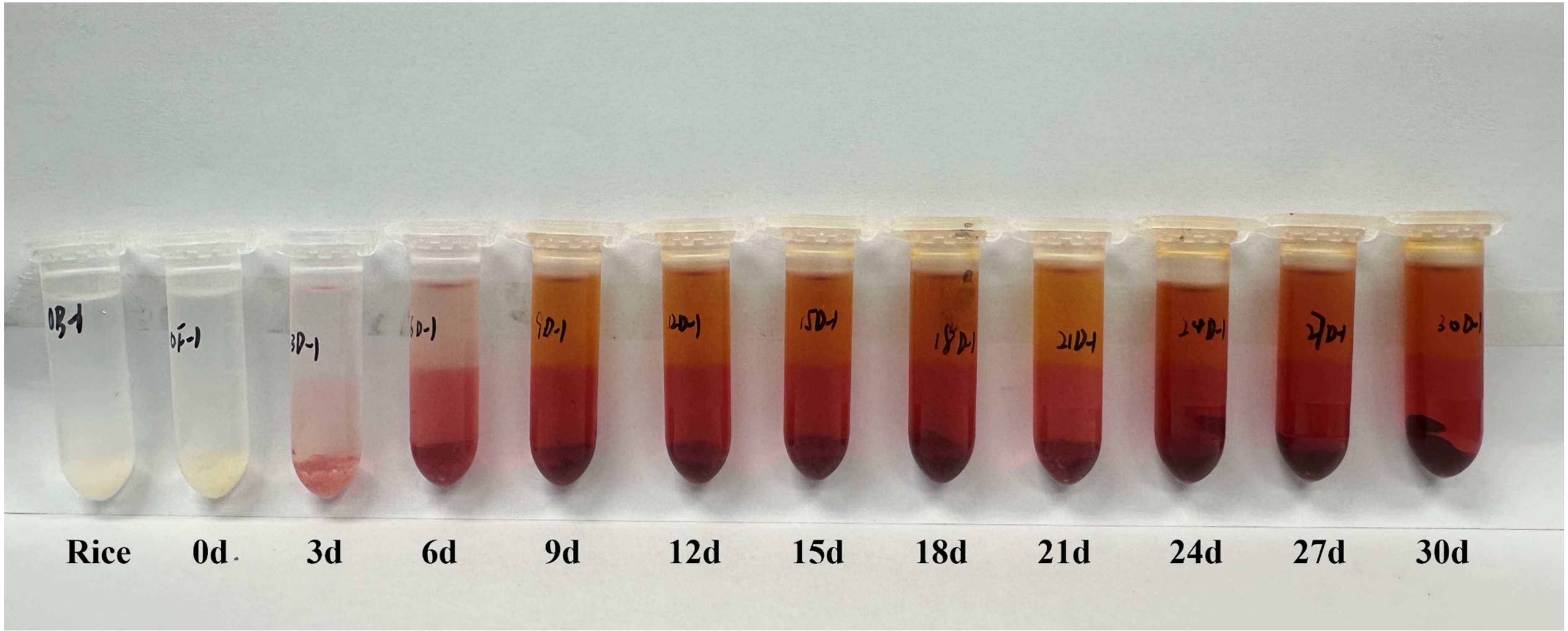

Figure S1. Extraction of rice products based on MTBE method.

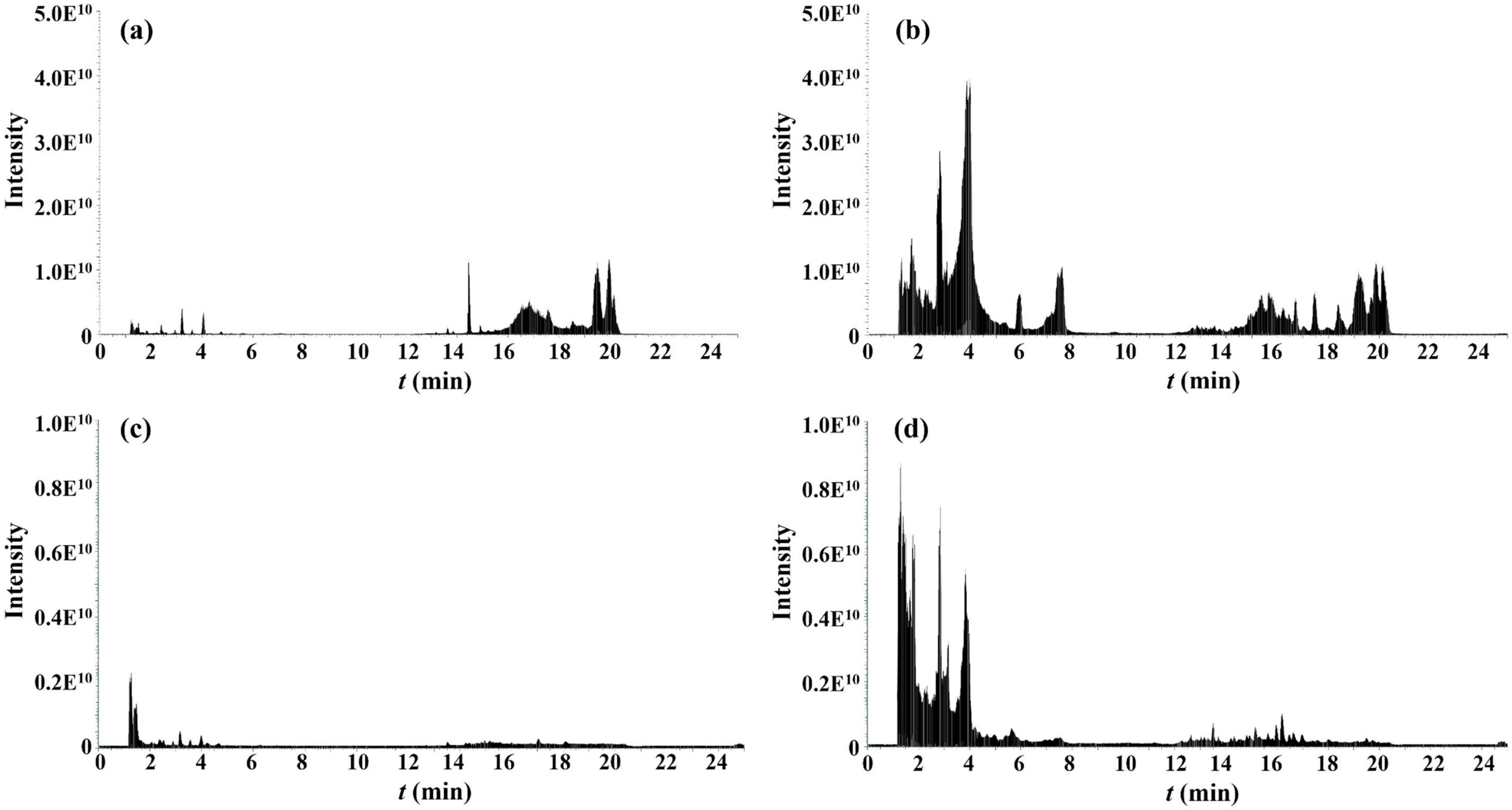

Figure S3. TIC of rice (a) and 30 days' fermentation (b) under ESI^+ ionization mode; rice (c) and 30 days' fermentation (d) under ESI^- ionization mode.
